# Indeterminate malignant potential glomus tumor of the esophagus: A case report

**DOI:** 10.1097/MD.0000000000042049

**Published:** 2025-05-30

**Authors:** Guifei Chen, Mingfa Wang, Yuchong Lin, Xueying Lin

**Affiliations:** a Department of Pathology, The Second Affiliated Hospital of Hainan Medical University, Haikou, Hainan, China; b Department of Interventional Oncology, Hainan Hospital of Guangdong Provincial Hospital of Traditional Chinese Medicine, Haikou, Hainan, China.

**Keywords:** diagnosis, differential diagnosis, esophagus, glomus tumor, immunohistochemistry, uncertain malignant potential

## Abstract

**Rationale:**

Glomus tumors of the esophagus are exceedingly rare neoplasms with poorly defined biological behavior and malignant potential. Their diagnostic ambiguity and management dilemmas necessitate detailed case documentation to refine clinical strategies.

**Patient concerns:**

A 62-year-old male presented with progressive 4-month dysphagia for solid foods, without weight loss or hematemesis.

**Diagnoses:**

Contrast-enhanced computed tomography identified a 50 mm × 23 mm mid-thoracic esophageal mass. Endoscopic ultrasound localized a hypoechoic muscularis propria-originating lesion. Histopathological examination postresection confirmed glomus tumor morphology, with immunohistochemical evidence of smooth muscle actin, H-caldesmon, and vimentin positivity. The tumor demonstrated a Ki-67 proliferation index of 8% without mitotic activity. Based on WHO criteria (deep visceral location, size >5 cm), it was classified as a glomus tumor of uncertain malignant potential (GT-UMP).

**Interventions:**

Complete surgical en bloc resection via right thoracotomy approach.

**Outcomes:**

No perioperative complications occurred. Serial imaging and endoscopic surveillance over 6 months showed no local recurrence or distant metastasis.

**Lessons:**

Esophageal GT-UMP diagnosis requires multimodal correlation: characteristic immunohistochemical profile (smooth muscle markers positivity) combined with WHO size/location criteria. The absence of recurrence in this large (5 cm) GT-UMP case challenges current size-based risk stratification, suggesting biological heterogeneity within this classification. Molecular profiling and extended (>5 years) follow-up are imperative to establish reliable prognostic markers for visceral glomus tumors.

## 1. Introduction

Glomus tumors originate from modified smooth muscle cells of the glomus body, predominantly found in the dermis or subcutaneous tissues, especially on the fingertips. While most glomus tumors are benign, those arising in deep visceral organs are rare and may exhibit malignant potential. Esophageal glomus tumors are exceptionally uncommon, with few cases documented in the literature.^[1]^ Clinically, these tumors often present with vague symptoms such as dysphagia or a sensation of a foreign body, which can easily be mistaken for other esophageal neoplasms like esophageal carcinoma or gastrointestinal stromal tumors (GISTs).^[2]^

Malignant glomus tumors form a rare subset, with diagnostic criteria including a tumor size over 2.0 cm, deep or visceral location, and histological features like nuclear pleomorphism or high mitotic activity.^[2]^ Tumors not fully meeting malignancy criteria but located in deep tissues or of significant size are classified as glomus tumors of uncertain malignant potential (GT-UMP).^[3]^ These tumors are unpredictable, possibly demonstrating aggressive behavior, complicating clinical management.

## 2. Case report

**Figure 1.**
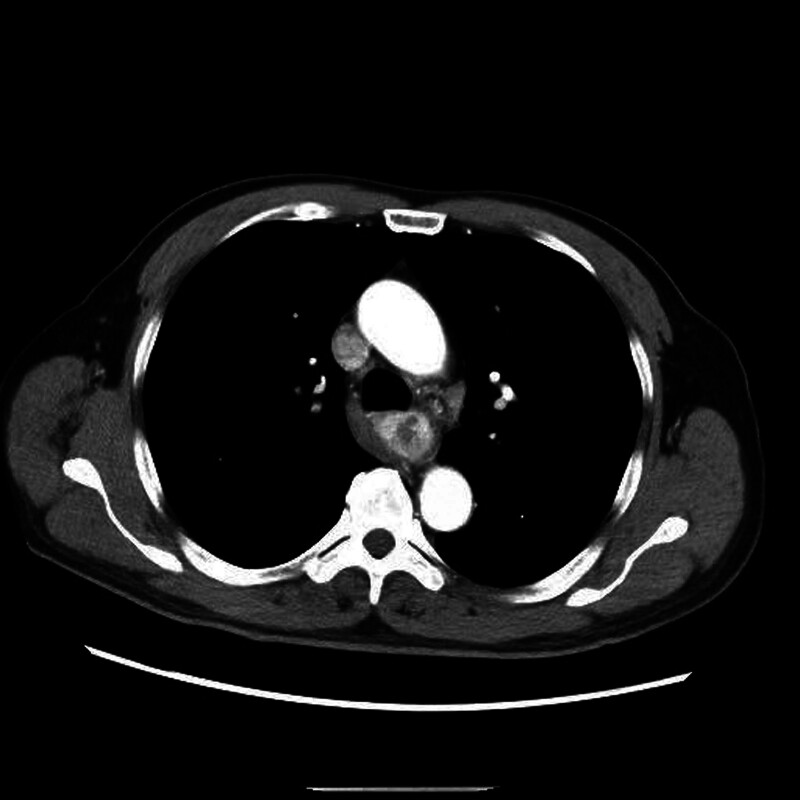
CT scan showing the mid-thoracic esophageal mass. CT = computed tomography.

A 62-year-old male presented with a 4-month history of experiencing a foreign body sensation during swallowing, particularly when consuming dry or solid food. Occasional mild obstruction was also reported. Physical examination was unremarkable. Computed tomography revealed eccentric thickening of the mid-thoracic esophageal wall, forming a well-defined 50 mm mass with a maximum thickness of 23 mm, causing esophageal lumen narrowing, Fig. 1. Contrast-enhanced imaging displayed uneven enhancement, with necrotic areas showing no enhancement. No lymph node enlargement was observed in the mediastinum.

Further evaluation via esophagogastroduodenoscopy was recommended. Endoscopic ultrasound revealed a smooth, semi-circumferential esophageal mass approximately 8 cm in length. The ultrasound showed a hypoechoic lesion with uneven echogenicity and clear margins, located within the muscularis propria, with the outer membrane intact. The initial diagnosis was an esophageal muscularis propria tumor, with GIST as a differential. Given that preoperative imaging suggested a deep-seated tumor location, and considering the significant risks associated with invasive procedures and biopsies for the patient, we opted for complete surgical resection to facilitate a definitive pathological diagnosis and assess potential malignancy, rather than performing a preoperative biopsy.

The patient underwent endoscopic resection of the esophageal tumor. Intraoperatively, an encapsulated mass was discovered near the mediastinum, occupying about one-quarter of the esophagus, measuring 8 cm × 8 cm. The tumor had a soft consistency, indistinct borders, and limited mobility. Both intraoperative and postoperative findings suggested a glomus tumor of the esophagus.

### 2.1. Pathological findings

**Figure 2.**
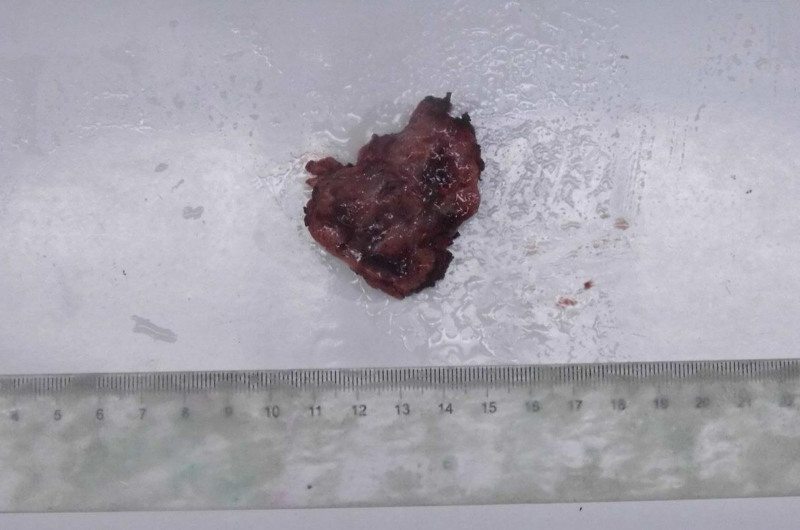
Gross view of the tumor section, showing nodular appearance with partial encapsulation. The cut surface appears gray-red, with visible areas of hemorrhage.

**Figure 3.**
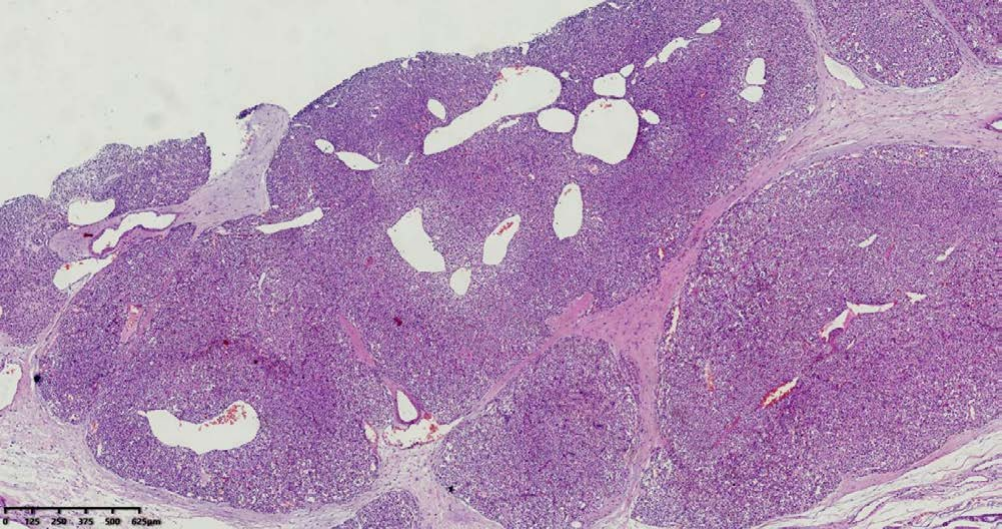
Histological sections show the tumor divided into nests by fibrous and smooth muscle septa, with abundant dilated vascular spaces within the tumor nests.

**Figure 4.**
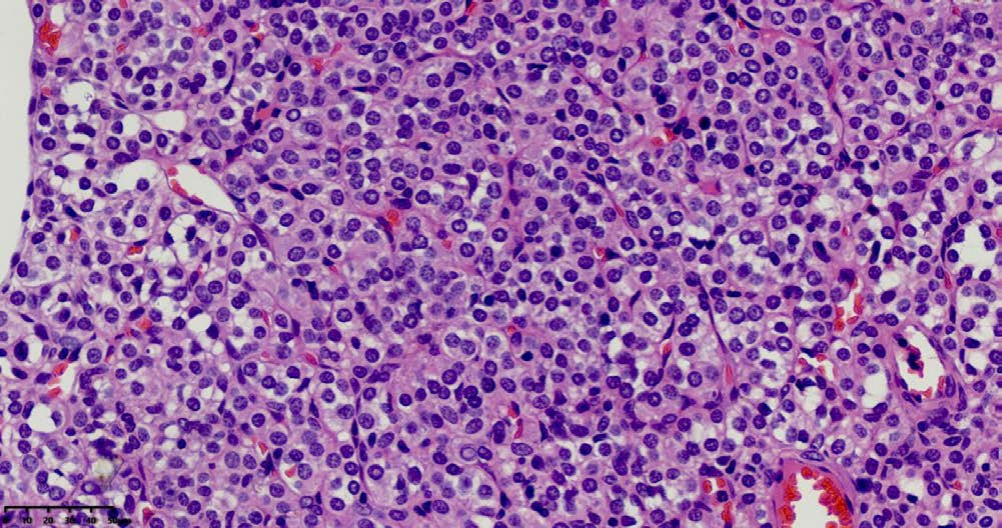
Tumor cells appear round with clear cytoplasm, surrounding capillary-like blood vessels.

Macroscopic examination revealed an irregular, gray-red mass measuring 4 cm × 3 cm × 2.5 cm, partially encapsulated, with a solid gray-red cut surface and areas of hemorrhage, Fig. 2. Microscopically, the tumor was composed of solid sheets and nests of round, uniform cells with clear or eosinophilic cytoplasm, Fig. 3. No mitotic figures were observed, and numerous small blood vessels were present, along with regions of hemorrhage, Fig. 4.

### 2.2. Immunohistochemical analysis showed the following results

**Figure 5.**
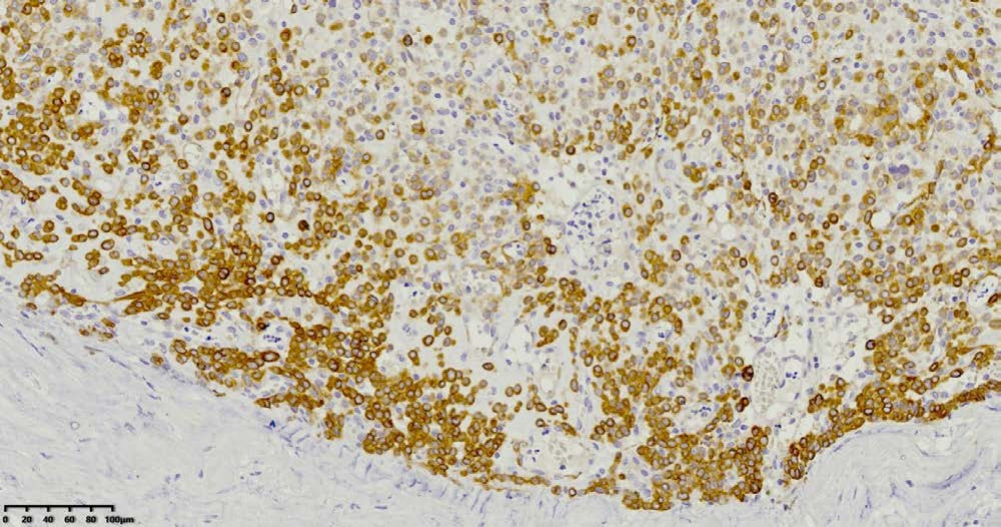
Focal expression of immunohistochemical marker H-cadherin using the Envision method.

CK (‐), smooth muscle actin (SMA) (+), S-100 protein (‐), vimentin (+), Calponin (focal +), Hcaldesmon (+), Synaptophysin (+), CD56 (+), CD117 (‐), discovered on GIST-1 (‐), CD34 (‐), Desmin (focal +), Ki-67 antigen (a marker for cell proliferation) (Ki-67) (8% positive), CD31 (vascular +), ETS-related gene (scattered +), factor VIII (‐), SRY-Box Transcription Factor 10 (‐), Fig. 5. These findings, combined with the clinical presentation, confirmed the final diagnosis of a glomus tumor of the esophagus with uncertain malignant potential.

## 3. Discussion

Glomus tumors originate from the glomus body, a variant of smooth muscle cells. Benign glomus tumors are typically found on body surfaces, especially in the dermis or subcutaneous tissues of the extremities. However, glomus tumors arising in deep visceral organs are rare. Malignant glomus tumors are even rarer, with Folpe^[1]^ establishing diagnostic criteria including: (1) tumor size > 2.0 cm; (2) deep or visceral tissue location; and (3) atypical mitotic figures, nuclear pleomorphism, or a mitotic rate of ≥5 per 50 high-power fields. The World Health Organization classifies malignant glomus tumors based on: (1) marked nuclear atypia (regardless of mitotic count) or (2) atypical mitotic figures. Glomus tumors that do not meet these malignancy criteria but show certain features such as: (1) superficial location without nuclear atypia but with mitotic activity > 5 per 50 high-power fields^[2]^; (2) tumor size > 2 cm; or (3) deep visceral location, are classified as GT-UMP.

We report a case of esophageal GT-UMP in a 62-year-old male. A 5 cm deep esophageal mass, initially misdiagnosed as GIST or leiomyoma, was confirmed as GT-UMP based on histopathology (uniform cells without mitotic activity) and immunohistochemistry (SMA/H-caldesmon positivity, Ki-67 index 8%). No recurrence at 6-month follow-up suggests biological heterogeneity. Long-term monitoring and molecular studies are needed to clarify prognostic determinants.

Reports in the literature suggest no significant gender differences in the incidence of GT-UMP, with the age of onset ranging from 15 to 74 years, and a median age of 58 years, which is older than the onset age for benign glomus tumors. GT-UMP typically occurs in the extremities, lungs, and stomach.^[3–5]^ Symptoms often result from the tumor’s mass effect, such as compression-induced cough, subcutaneous pain in the extremities, or submucosal gastric lesions. Esophageal glomus tumors are rare, and their clinical presentation often includes dysphagia or, in some cases, the incidental finding of an esophageal mass during unrelated examinations. In this case, the patient experienced a foreign body sensation during swallowing, prompting further endoscopic evaluation that revealed the esophageal mass.

While rare, many reported esophageal glomus tumors exhibit malignant or potentially malignant behavior. Cases of malignant esophageal glomus tumors typically occur in patients over 40, with dysphagia being the primary symptom. This case, characterized by dysphagia, was diagnosed with computed tomography, endoscopic ultrasound, and pathology, treated with surgical resection, and resulted in a favorable outcome with no complications or recurrence. While it shares similarities with some reported cases (e.g., dysphagia as a symptom, surgical resection as treatment), it differs from others that were asymptomatic, used additional diagnostic tools like molecular testing, employed alternative treatments, or had poorer prognoses,^[6–11]^ Table 1. These tumors are generally larger than 2 cm in diameter, and histological examination often reveals nuclear atypia. Other signs of malignancy, such as tumor ulceration, peripheral infiltrative growth, and vascular or neural invasion, have also been observed.^[6,7,12,13]^ Some cases reported pulmonary metastasis,^[6]^ while others involved lymph node metastasis.^[13]^ One instance of an esophageal glomus tumor with uncertain malignant potential was found as an asymptomatic esophageal mass.^[8]^ Compared to gastric glomus tumors, esophageal glomus tumors appear to have a higher likelihood of aggressive behavior, particularly for tumors larger than 5 cm, which are more prone to malignant transformation and warrant closer monitoring.^[14]^ In one case of malignant esophageal glomus tumor, the initial resection specimen showed no nuclear atypia, but further sampling from different tumor regions revealed malignant areas.^[6]^ This highlights the importance of cautious diagnosis, especially for biopsy and large resection specimens. Comprehensive sampling from multiple regions of the tumor is essential for accurate diagnosis.

**Table 1:** Summary of reported esophageal glomus tumor cases and comparison with this case.

Case source	Age/sex	Symptoms	Tumor Size (cm)	Location	Diagnostic method	Treatment	Molecular features	Outcome (follow-up)
This case	62/M	Dysphagia (4 months)	4.0 × 3.0 × 2.5	Mid-esophagus	Pathology + IHC (SMA+, Ki-67 8%)	Surgical resection	None	No recurrence (6 months)
Xiao et al^[6]^	57/F	Dysphagia, weight loss	5.0	Mid-esophagus	Biopsy + IHC (SMA+)	Palliative RT + chemotherapy	MIR143::NOTCH2 fusion	Transitioned to hospice (4 months)
Birkness-Gartman et al^[7]^	65/M	Dysphagia	4.5	Esophagus	Pathology + IHC (SMA+)	Surgery	NOTCH3 rearrangement	Alive with disease (9 years)
Marcella et al^[8]^	30/M	Asymptomatic	2.0 × 1.8	Mid-esophagus	EUS + IHC (SMA+, Ki-67 12%)	STER	None	No recurrence (1 year)
Moore et al^[9]^	80/M	Asymptomatic	2.5	Mid-esophagus	EUS-FNA (SMA+)	Observation	None	NA
Pancsa et al^[10]^	20/M	Dysphagia	4.0	Esophagus	Surgery + IHC (SMA+)	Surgery + chemotherapy	CARMN::NOTCH2 fusion	Died of disease (2 years)
Matsumoto et al^[11]^	45/M	Asymptomatic	1.5	Lower esophagus	EUS-FNA + IHC (SMA+)	Thoracoscopic enucleation	None	No recurrence (1 year)

EUS = endoscopic ultrasound.

The immunophenotypic and molecular characteristics of esophageal glomus tumors with uncertain malignant potential closely resemble those of benign glomus tumors. Reported cases show expression of markers like SMA, H-caldesmon, Calponin, vimentin, and type IV collagen. In this case, the esophageal tumor’s immunophenotype was consistent with previously reported cases and also expressed synaptophysin with a Ki-67 index below 10%. Some authors have proposed that the expression of neuroendocrine markers may be related to tumor location, suggesting that glomus tumors in specific regions may have partial neuroendocrine functions. A higher Ki-67 index could be linked to an increased risk of recurrence.^[2,15,16]^

Given the rarity of esophageal glomus tumors and their nonspecific clinical presentation, preoperative diagnosis is often difficult. The final diagnosis depends mainly on pathological features and immunohistochemistry, making adequate tissue sampling crucial. Differential diagnosis should consider submucosal gastrointestinal stromal tumors (GISTs), leiomyomas, sarcomas, and neuroendocrine tumors, which can be excluded based on histological, immunohistochemical, and molecular findings. Due to the rarity of these tumors, standardized guidelines for staging, treatment, or follow-up are lacking. Surgical resection remains the primary treatment for localized tumors, while adjuvant radiotherapy or chemotherapy shows limited efficacy. In this case, the patient was followed up at 3 months and 6 months postoperatively, and the current follow-up status is also available. The patient is currently in good condition, with no significant recurrence or complications observed. Additionally, we have established a long-term follow-up plan for the next 1 to 2 years.

This study has several limitations. First, as a single-center case report, the small sample size restricts the generalizability of the findings. Second, the postoperative follow-up period remains relatively short (6 months), and long-term prognostic data (e.g., recurrence or metastasis beyond 2 years) are unavailable. Third, molecular analyses (e.g., gene fusion or mutation profiling) were not performed due to the tumor’s rarity, potentially missing molecular signatures linked to malignant potential. Additionally, the lack of preoperative biopsy may introduce bias in correlating imaging and histopathological assessments. Future multi-center studies with extended follow-up periods and integrated molecular profiling are warranted to clarify the biological behavior and prognostic determinants of esophageal glomus tumors.

## Acknowledgments

The authors are grateful to the patient who participated in this study.

## Author contributions

**Conceptualization:** Xueying Lin.

**Data curation:** Guifei Chen, Mingfa Wang, Yuchong Lin.

**Funding acquisition:** Xueying Lin.

**Investigation:** Guifei Chen, Yuchong Lin.

**Methodology:** Guifei Chen, Mingfa Wang, Yuchong Lin.

**Project administration:** Xueying Lin.

**Resources:** Mingfa Wang.

**Writing – original draft:** Guifei Chen, Xueying Lin.

**Writing – review & editing:** Guifei Chen, Xueying Lin.
